# Microdissection is the best way to perform sperm retrieval in men with non-obstructive azoospermy? | *Opinion: Yes*

**DOI:** 10.1590/S1677-5538.IBJU.2018.06.02

**Published:** 2018

**Authors:** Renato Fraietta

**Affiliations:** 1 Setor Integrado de Reprodução Humana, Universidade Federal de São Paulo, São Paulo, SP, Brasil

**Keywords:** Azoospermia, Microdissection, Sperm Retrieval, Fertility

Non-obstructive azoospermia (NOA) is the diagnosis of one percent of all men and 10% of men complaining about infertility (1, 2). All NOA patients should be evaluated with complete history and physical examination, with genetic testing (karyotype analysis and Y chromosome microdeletion testing) being offered and performed, which will identify the causes of NOA in up to 17% of men (3, 4). Hormonal profile is also important as up to 47% of men that have impaired spermatogenesis with NOA were found to have hypogonadism (4,5).

For this situation and after the breakthrough of intracytoplasmic sperm injection (ICSI) in 1992, a man with NOA can be a genetic father if it is possible to obtain viable sperm directly from his testis. Classically, there are two ways to perform it: percutaneously (TESA - TEsticular Sperm Aspiration) through fine needle aspiration (FNA) - which is dependent on a small amount of sampling - or surgically. Nowadays, there are two basic surgical techniques to retrieve sperm: conventional TEsticular Sperm Extraction (cTESE) and testicular microdissection (or also micro-TESE), which is the topic of this session Difference of Opinion.

Until recently, cTESE was considered gold standard for retrieving sperm in men with NOA (6). During a cTESE procedure, the testis is exposed through a small incision and one or multiple biopsies - more commonly - are taken randomly under direct sight (6). According to Donoso et al., cTESE has an average retrieval rate around 50% in NOA men (6, 7). This procedure is performed under general or regional anesthesia in a daily-basis clinical center.

Testicular microdissection was first introduced in 1999 (6, 8). Micro-TESE consists of an equatorial testicular opening in order to retrieve engorged seminiferous tubules that are more likely to contain active spermatogenesis, with the use of a surgical microscope (6). This procedure requires admittance to a hospital and general or regional anesthesia.

## Microdissection-TESE (micro-TESE) versus conventional TEsticular Sperm Extraction (cTESE)

Some authors have shown that microdissection is the best way to perform sperm retrieval in men with NOA, not only in terms of sperm retrieval (SR) but also when considering complications to the technique itself. Three recent studies are more representative of this subject, a systematic review and meta-analysis (2), a systematic review (6) and a review (4), which deserve the following considerations:

## Sperm retrieval

Bernie et al. performed a systematic review and meta-analysis that identified fifteen studies of 1,890 total patients, published between 1997 and 2012 (2). In a direct comparison of cTESE to micro-TESE, the unadjusted SR was 35% for cTESE (95% CI 30%-40%) and 52% for micro-TESE (95% CI 47%-58%) (2). Performance of micro-TESE was 1.5 times more likely (95% CI 1.4-1.6) to result in successful SR as compared with cTESE (2).

The aim of the study by Deruyver et al. was to compare the outcome of cTESE with micro-TESE through a systematic review of the literature comparing these two methods (6). Primary outcome was sperm retrieval rate in the micro-TESE group and in the cTESE group. Secondary outcome was other clinical predictors of positive sperm retrieval. Seven studies were included: two were prospective, non-randomized studies (Schlegel, 1999; Amer et al., 2000) (8, 9). Three studies were retrospective (Okada et al., 2002; Tsujimura et al., 2002; Ramasamy et al., 2005) (10-12) and the two remaining studies were pseudo-randomized controlled studies (Colpi et al., 2009; Ghalayini et al., 2011) (13, 14). The SR in the cTESE group ranged from 16.7 to 45% and in the micro-TESE group from 42.9 to 63%. Five of the seven studies showed a significant difference (p < 0.05) in favor of micro-TESE.

Sertoli cell only syndrome, a histological condition characterized by absence of germ cells with only normal Sertoli cells lining the seminiferous tubules predicted a significant better result in the micro-TESE group according to two studies (Okada et al., 2002; Ghalayini et al., 2011) (10, 14). Results ranged from 22.5 to 41% in the micro-TESE and from 6.3 to 29% in the cTESE group. No safe clinical predictors of sperm retrieval were demonstrated for both procedures (6).

In their review, Schlegel et al. reported an overall experience result with micro-TESE of 52% (607/1176) sperm retrieval rate including post-chemotherapy, Klinefelter's syndrome, cryptorchidism and AZFc deletion patients (4). According to the authors, for men who undergo cTESE and fail to have sperm retrieval, a repeat cTESE causes further testicular damage with limited success (4). In case of a failed cTESE, a salvage micro-TESE can be offered and sperm retrieval is possible in 45% of times (4, 15).

## Complications

Aside from better sperm retrieval results, micro-TESE represents the technique with lower chances of complication. Comparing with micro-TESE, possible complications after cTESE are low but include loss of significant amount of testicular tissue, hematoma, inflammatory changes and permanent devascularization (6, 16). With this in mind, a possible advantage of the micro-TESE technique is a better identification of sub-tunical vessels and, as a consequence, reducing the risk of devascularization (6).

Although fewer sonographic complications may occur after micro-TESE, clinical complication rate between both procedures seems not to differ (6). Three of the included studies systematically compared the sonographic changes at different months of follow-up (Amer et al., 2000; Okada et al., 2002; Ramasamy et al., 2005) (9, 10, 12). Hematoma was less frequent in the microTESE group after 1 and 3 months. Fibrosis and decreased testicular volume (> 2 mL) were also less frequent in the micro-TESE group at 6 months. In the study of Okada et al., a significant decrease in serum testosterone after 6 months was observed in two patients in the cTESE group, whereas none occurred in the micro-TESE, although this was not statistically significant (9). Ramasamy et al. reported no significant difference in return to baseline testosterone levels between the two procedures (12).

## Limitations

Histological findings from the testis of men with NOA vary and may include Sertoli cell only syndrome, maturation arrest (precocious or late) or hypospermatogenesis.

According to Bernie et al., the difference in sperm processing, the patient heterogeneity that exists in the population of men diagnosed with NOA, and the practice patterns and differing surgeon skill levels often make it difficult to know the true differences between the extremely varied SRs for these procedures (2).

The choice of SR technique to perform in a man with NOA is not only dependent on the predicted SR, but also should be oriented by previous procedure history, knowledge of testicular pathology, potential for postoperative complications, cost of the procedure, and knowledge and skill of the surgeon (2).

A considerable number of cases of surgeon experience are necessary to reach a relative plateau level of SR, and at least 50 cases are needed to pass the steepest portion of the learning curve. Subtle continued increases in SRs appear to occur as a surgeon exceeds experience with more than 500 micro-TESE procedures (4).

When it comes to duration of the procedure and cost, in comparison with cTESE, micro-TESE procedures are much more time-consuming and require the use of an operating microscope, which increases the cost of the technique (6).

In the review published by Schlegel et al., the mean operative time was 1.8 h (range 0.5-6.6 h) for successful micro-TESE and 2.7 h (range 0.8-7.5 h) for attempts in which sperm were not found (4). Besides, a higher number of embryologists is necessary to look for sperm during the whole attempt.

## Final considerations

As already known, it is very difficult to consider pregnancy rates after intervening in the male factor because they involve female potential impact which is not always evaluated in the studies. According to Bernie et al., because of incomplete reporting, analysis of other patient characteristics and outcomes (e.g., pregnancy) was not possible in their work (2). On the other hand, Schlegel et al. reported a pregnancy rate of 48% out of 1,414 overall experience NOA men cycles (4).

Furthermore, so far no clinical studies have compared birth rate between cycles using spermatozoa retrieved through cTESE and micro-TESE procedures (6).

A reason for bias in the studies that compare cTESE and micro-TESE is the fact that the latter is usually indicated in more severe situations.

Therefore, SR through micro-TESE may actually be artificially lowered by the fact that many men undergoing micro-TESE have failed a previous TESA or cTESE, suggesting that if all men treated with NOA were randomized from the very beginning, the difference between micro-TESE and cTESE might be even more pronounced (2).

## CONCLUSIONS

Recent studies have shown that testicular microdissection is the best way to retrieve sperm from men with non-obstructive azoospermia.

Although micro-TESE provided the highest SR in these analyzes, the authors do not necessarily recommend that this be the only method of SR performed in men with NOA. Studies with standardized reporting are necessary that may allow for a better understanding of the true differences in SRs for each technique in men with NOA, as well as help to guide when it may be reasonable to perform a particular procedure (2).
